# Superior haplotypes towards development of low glycemic index rice with preferred grain and cooking quality

**DOI:** 10.1038/s41598-021-87964-8

**Published:** 2021-05-12

**Authors:** Ramchander Selvaraj, Arun Kumar Singh, Vikas Kumar Singh, Ragavendran Abbai, Sonali Vijay Habde, Uma Maheshwar Singh, Arvind Kumar

**Affiliations:** 1 IRRI South Asia Hub (IRRI-SAH), ICRISAT Campus, Patancheru, Hyderabad, India; 2 South-Asia Regional Centre (SARC), International Rice Research Institute (IRRI), Varanasi, India; 3 Leibniz Institute of Plant Genetics and Crop Plant Research (IPK), Gatersleben, Germany

**Keywords:** Biotechnology, Plant sciences

## Abstract

Increasing trends in the occurrence of diabetes underline the need to develop low glycemic index (GI) rice with preferred grain quality. In the current study, a diverse set of 3 K sub-panel of rice consisting of 150 accessions was evaluated for resistant starch and predicted glycemic index, including nine other quality traits under transplanted situation. Significant variations were noticed among the accessions for the traits evaluated. Trait associations had shown that amylose content possess significant positive and negative association with resistant starch and predicted glycemic index. Genome-wide association studies with 500 K SNPs based on MLM model resulted in a total of 41 marker-trait associations (MTAs), which were further confirmed and validated with mrMLM multi-locus model. We have also determined the allelic effect of identified MTAs for 11 targeted traits and found favorable SNPs for 8 traits. A total of 11 genes were selected for haplo-pheno analysis to identify the superior haplotypes for the target traits where haplotypes ranges from 2 (*Os10g0469000*-GC) to 15 (*Os06g18720*-AC). Superior haplotypes for RS and PGI, the candidate gene *Os06g11100* (H4-3.28% for high RS) and *Os08g12590* (H13-62.52 as intermediate PGI). The identified superior donors possessing superior haplotype combinations may be utilized in Haplotype-based breeding to developing next-generation tailor-made high quality healthier rice varieties suiting consumer preference and market demand.

## Introduction

Rice (*Oryza sativa* L.) is a major source for carbohydrate of more than 50% of the global population. In recent times, an increase in living standards has created awareness among the peoples all over the world for consumption of superior quality rice to improve their health status^1^. Thus, the presence of premium grain quality in rice is one of the crucial determinant factors for all the stakeholders including breeders, producers and consumers^2^. Currently, more efforts have been put forth to improve the rice quality traits by utilizing the key allelic variants from the germplasm variation to meet quality rice demand of consumers and potential markets. But, what are the key grain quality determinants? They are broadly classified in to, milling, appearance, cooking/eating parameters and nutritional quality. Among these, the later two are associated with the end-users. The physicochemical characters which determine the cooking and eating quality are amylose content (AC), gel consistency (GC) and gelatinization temperature (GT) or alkali spreading value (ASV). Generally, rice with intermediate amylose, GT and soft to medium gel consistency are preferred by consumers in South Asia^3^. Apart from quality, these days people have more concerns about their health, and prefer low glycemic index rice varieties which could be safe for diabetics and obesity. Hence, grain quality improvement of rice together with the lower glycemic index are of vital importance for the rice breeders in the current scenario of increasing diabetic population all over the world, especially in the Asia. Low GI diets effectively prevent type II diabetes^4,5^ and consumption of high amylose class rice varieties with soft-textured on cooking might be an alternative for intermediate amylose rice varieties especially for those who are suffering from type II diabetes^6^. The most critical factor responsible for breeding low glycemic index (GI) rice is resistant starch (RS). It is the starch portion resistant to enzyme hydrolysis that escapes the small intestine and enters the large intestine where it gets fermented and slowly releases glucose to the bloodstream. Generally, it has been reported that increased level of RS content in rice grains lowers the GI value ensuring a negative correlation with amylose content^7^. Recently low-to-intermediate RS phenotypic variations in rice panel were identified, resulting novel RS associations to numerous genes associated with amylopectin biosynthesis and degradation^7^.

In any crop species, genetic diversity plays important role in breeding elite varieties. Identification of favorable alleles^8^ and its superior haplotype of the various genes associated with traits linking to cooking and eating quality of rice are pre-requisite of breeding to develop healthier rice. Nowadays, with the improvement of high-throughput sequencing technologies with reduced cost makes genome wide association studies (GWAS) as on of the prominent techniques to identify marker-trait associations (MTAs). Research on the glycemic index identified a novel association of candidate loci *Os05g03600* reporting intermediate to high GI variations^9^. Another hotspot on chromosome 6 was found to include *GBSSI*, hydrolase genes and genes involved in signaling and chromatin modification with differential methylation patterns in *GI6.1* region. Alternative splicing of *GBSSI* promoter region resulted in intermediate to high GI variations. Novel SNP associations on chromosomes namley 2, 5, 6 and 11 has been reported and these SNPs influences the final viscosity variations but no significant association with GI^9^. Besides, the predicted glycemic index (PGI) is estimated by *in-vitro* enzymatic action of starch digestion leads to hydrolysis and the glycemic index (GI) estimated *in-vivo*, which requires human clinical evaluation of the two to three hours of blood glucose response after food intake. Thus, it is more time consuming and resource demanding. The *in-vitro* digestion methods have been developed to measure the starch hydrolysis index and it is used to calculate PGI using the formula developed^10^. Significant positive association (*r* = 0.946) between pGI and GI of rice samples by using bread as the reference^11^.

Recently, 11 candidate loci controlling grain quality traits has been identified that are involved in the starch biosynthesis^12^. Nine MTAs were identified for seven QTLs namely *GS3*, *TUD*, *qGRL7.1*, *qPGWC7*, *qGL3.4*, *qGW1.1* and *qGW10.2* controlling quality traits^13^. Numerous genes/QTLs were identified for grain number and grain length viz., *GW2, GIF1, qSW5, GS3, GS5, qGL3, GW8, GS6, GS2, GL7/GW7, OsMA PK6, GLW7* and *GAD1*^14–26^. QTLs controlling grain size have also been detected via genetic mapping and association studies^24,27–32^. Starch is made upof two components namely amylose and amylopectin. Amylose, an important parameter affecting cooking and eating quality of rice directly involved in changing the grain texture by absorbing water on cooking. However, the difference in the amylopectin structure of similar AC rice explained variations in the textural qualities of cooking. It is a well-established fact that the waxy (*Wx*) gene, *granule-bound starch synthase* (*GBSSI*) located on chromosome 6 and genomic regions surrounding the gene are highly diverse^9,33^. The *Alk* locus carrying *SSIIa* (*starch synthase IIa*) mapped on chromosome 6 confers alkali spreading to determine the gelatinization temperature of milled rice^34^. There are several other starch synthesis-related genes involved namely, starch debranching enzymes, starch branching enzyme soluble starch synthase with a predominant role in controlling eating and cooking properties of rice^35^. Two QTLs for resistant starch in rice *qRS7-1* and *qRS7-2* (chromosome 7), explaining phenotypic variance from 7.6 to 17.3%, have been reported^36^.

**Figure 1:**
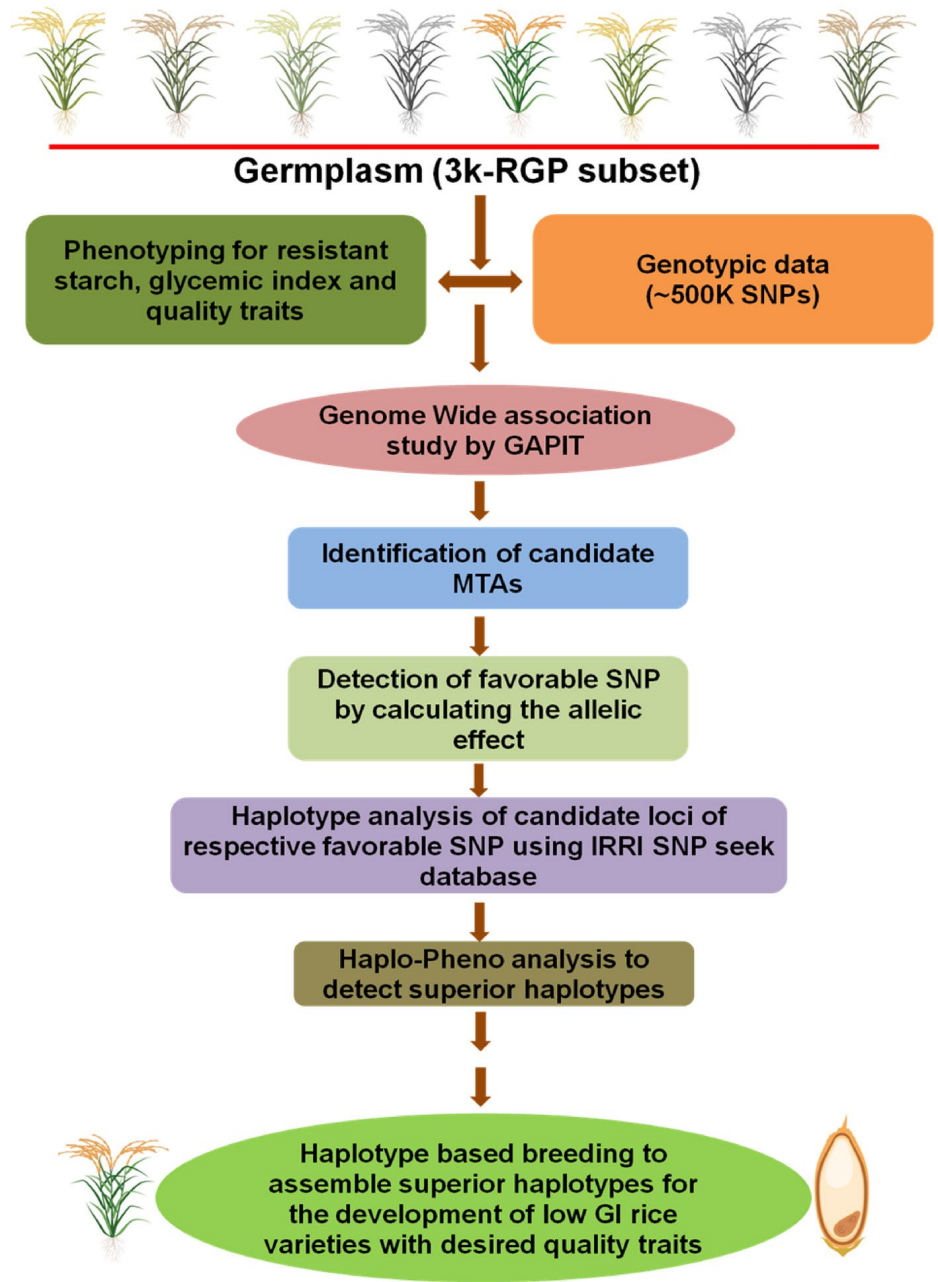
Overall methodology flow of the study to unravel the SNP associations, identifying favorable SNPs, locating the candidate genes and superior haplotype identification in transplanted rice (TPR) by haplotype based breeding to assemble superior haplotypes to design the rice varieties suitable for diabetics with improved grain quality parameters.

Rice grain quality is one of the major determinants in selecting parents for any breeding program and the genetic constitution of the genotypes determine the effect of prevailing environmental conditions on the developmental process involved in seed formation and maturation. The good quality grain is an important factor influencing its acceptance by consumers and thus, is one of the major traits in rice breeding to withstand marketability in trade and commerce. However, one of the significant challenges to date has been the lack of extensive knowledge of the genetic and molecular basis of several grain quality traits including GI. The availability of 3000 rice genomes (3 K-RGP) offers opportunities for harnessing haplotype diversity for GI along with other critical quality-related genes and enhances the possibility for the identification of ideal haplotypes for deployment in rice breeding. The current study was undertaken with the objective to identify the candidate genes and their superior haplotypes associated with RS, PGI and nine major grain quality traits across the 3 K-RGP subset under transplanted (TPR) situation (Fig. 1) and explore the possibility of developing healthy rice varieties with preferred grain quality by assembling superior haplotypes via *haplotype-based breeding*.

## Results

### Trait variation for resistant starch, glycemic index and grain and cooking quality

The subset of 3 K RGP was extensively phenotyped to study the variations for RS, PGI along with nine other grain quality-related traits. The results revealed significant variations among the accessions (Table S1). The RS content ranged from 0.57 to 10.00%, whereas, the PGI ranged from 52.91 to 99.94. Significant variation was also observed for the other quality traits-like AC, LBR, LER, ASV and GC that play an important role in the selection of desirable quality of rice varieties in the view of consumer preference. In our study, AC ranged from 12 to 33%, KL in the range of 4.60 to 7.7 mm, KB ranged from 1.80 to 3.00 mm, LBR in the range of 1.58 to 3.60, ASV from 1 to 7 score, LER ranged between 1.02 to 1.96 and GC in the range of 14.50 to 100 mm.

### Correlation among grain quality traits

**Figure 2:**
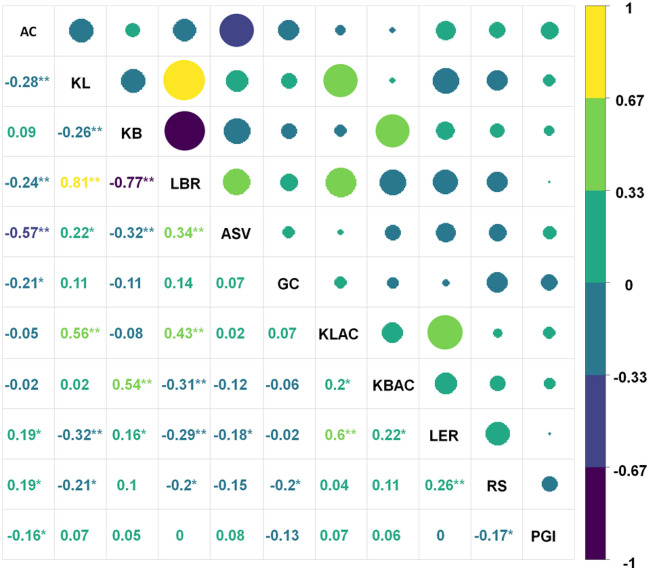
Investigation on correlation coefficients explaining associations among predicted glycemic index and resistant starch with rice quality parameters under transplanted rice system. AC—Amylose content, KL—Kernel length, KB—Kernel breadth, LBR—Length/ Breadth ratio, ASV—Alkali spreading value, KLAC—Kernel length after cooking, KBAC—Kernel breadth after cooking, LER—Linear elongation ratio, GC—Gel consistency, RS—Resistant starch, PGI—Predicted glycemic index, TPR—Transplanted rice. * Significance at *p*-value < 0.05, ** Significance at *p*-value < 0.01.

Association among the traits was observed. RS and PGI showed significant negative correlations. The important quality trait AC showed a significant positive (r = 0.19*, *p*-value-0.048) and negative correlation (r = − 0.16*, *p*-value-0.049) with RS and PGI, respectively whereas; it showed a negative significant relationship with ASV and GC. KL had a significant positive correlation with LBR and KLAC whereas it was negatively correlated with KB (Fig. 2).

### Genome-wide association study (GWAS) of targeted traits

**Figure 3:**
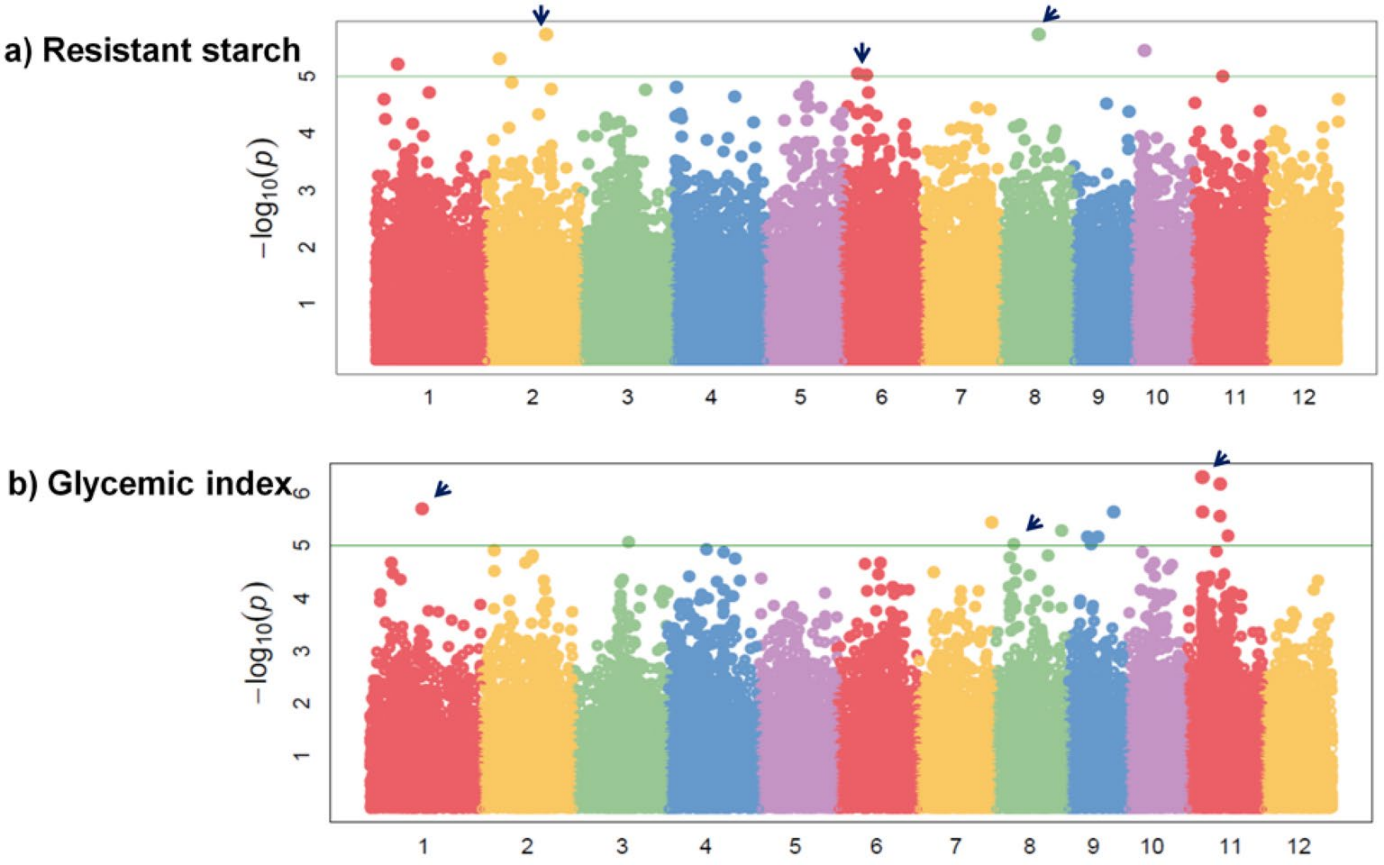
Genome wide association studies—Manhattan plot showing peak associations for resistant starch and predicted glycemic index in (**a**) Peak association for resistant starch on chromosome 2, 6 and 8 in transplanted rice, (**b**) Peak association for PGI on chromosome 1, 8 and 11 in transplanted rice.

Single locus GWAS (MLM) was performed using the phenotypic data with approximately more than 0.5 million (500 K) SNPs of concerned accessions and the peak associations were predicted based on considering the value of PVE (phenotypic variance explained) with suggestive significant *p*-value < 0.00001 (− log_10_ (*p*) > 5). In general, modification of bonferroni correction significant threshold value to control the false positive rate in a single-locus GWAS are so conservative that some associated SNPs may be eliminated. To avoid this problem multi-locus GWAS of mrMLM was also conducted to confirm the MTAs with the LOD score of greater than 3. A set of 41 causatives MTAs were detected for all the investigated quality traits in MLM (Table 1). In MLM, single MTA was identified for KL, KBAC, L/B ratio and LER, and up to nine MTAs for AC, RS and PGI. We identified nine MTAs associated with RS located on chromosomes 1, 2, 6, 8 and 11 with PVE ranging between 15.92 to 19.31% (Fig. 3). For PGI, nine MTAs were identified on chromosomes 1, 3, 7, 8, 9 and 11 with PVE ranging between 23.47 to 17.77% (Fig. 3). For AC, nine MTAs were detected on chromosomes 2, 6, 8, 10 and 11, respectively with the PVE ranging between 14.38 to 17.96% (Fig. S1). For KL, one MTA was identified on chromosome 11 with the PVE of 11.87% (Fig. S2). For KB, 4 MTAs were identified on chromosome 2, 3 and 5 with a PVE of 14.48, 14.79, 15.35 and 21.48%, respectively (Fig. S3). LBR recorded an MTA on chromosome 5 with the PVE of 16.99% (Fig. S4). For ASV, an indicator for GT, one peak was detected on chromosome 6 with a PVE of 25.53% (Fig. S5). Three MTAs on chromosome 1, 5 and 12 were detected for KLAC with PVE of of 23.55, 24.17 and 18.05%, respectively (Fig. S6). For KBAC only one association was detected on chromosome 8 with the PVE of 18.35 (Fig. S7). For LER, one MTA was identified on chromosome 9 explaining PVE of 16.69%. Two MTAs for GC on chromosome 10 and 7 with the PVE of 18.39 and 18.11% were detected in TPR (Fig. S8).

**Table 1:** GWAS for grain quality traits, RS content and predicted glycemic index by single locus (MLM) and multi-locus model (mrMLM).

Trait	SNP ID	MLM		mrMLM		Gene ID (RAP)	Gene function
		P value	PVE (%)	LOD Score	PVE (%)		
AC	S8_10622940	1.21E-06	17.96	4.64–5.63	1.85–4.44	*Os08g0276000*	transmembrane 9 superfamily member, putative, expressed
	**S6_6896749**	**6.05E-06**	**15.43**	**4.74–5.82**	**11.52–21.83**	***Os06g0232700***	**transposon protein, putative, unclassified, expressed**
	S8_10166593	7.33E-06	15.13	3.88–4.65	1.64–3.61	*Os08g0266700*	Rad21 / Rec8 like protein, putative, expressed
						*Os08g0267050*	expressed protein
	**S11_28809145**	**8.24E-06**	**14.95**	**4.56–10.66**	**9.26–11.50**	***Os11g0704000***	**selT/selW/selH selenoprotein domain containing protein, expressed**
	S2_1904604	9.63E-06	14.71	4.52–6.34	3.61–11.47	*Os02g0135800*	WD domain, G-beta repeat domain containing protein, expressed
	S10_5085990	1.02E-05	14.61	3.17–4.35	3.94–13.37	*Os10g09430*	retrotransposon protein, putative, Ty3-gypsy subclass, expressed
	S6_10612428	1.12E-05	14.47	5.12–8.70	7.70–25.81	*Os06g18710*	retrotransposon protein, putative, unclassified
						*Os06g18720*	retrotransposon protein, putative, Ty3-gypsy subclass, expressed
	S8_12286113	1.19E-05	14.39	3.56–4.30	1.70–3.86	*Os08g0299700*	OsFBO20—F-box and other domain containing protein, expressed
	S2_2474176	1.20E-05	14.38	4.65–6.75	4.20–13.68	*Os02g05180*	retrotransposon protein, putative, Ty3-gypsy subclass, expressed
KL	S11_28909306	6.64E-06	11.87	3.55–3.95	1.05–1.22	*Os11g0706100*	alpha-hemolysin, putative, expressed
						*Os11g0706500*	expressed protein
KB	**S5_5361877**	**1.19E-07**	**21.48**	**5.32–8.12**	**1.01–5.29**	***Os05g09510***	**hypothetical protein**
						*Os05g0187500*	IQ calmodulin-binding motif family protein, expressed
	S3_13543674	5.46E-06	15.35	3.68–4.18	1.00–1.01	*Os03g23860*	retrotransposon protein, putative, Ty3-gypsy subclass, expressed
	S3_19893628	7.87E-06	14.79	4.17–5.04	1.01–1.04	*Os03g35870*	retrotransposon protein, putative, Ty3-gypsy subclass, expressed
	S2_13138765	9.63E-06	14.48	33.55–4.38	1.01–1.06	*Os02g0326500*	expressed protein
LBR	S5_5361877	8.33E-08	16.99	6.30–9.12	1.01–12.26	*Os05g09510*	hypothetical protein
						*Os05g0187500*	IQ calmodulin-binding motif family protein, expressed
ASV	**S6_6711302**	**6.31E-08**	**25.53**	**7.87–18.86**	**10.55–27.19**	***Os06g0229000***	**OsFtsH6 FtsH protease, homologue of AtFtsH6, expressed**
						*Os06g12380*	expressed protein
KLAC	**S5_28169331**	**5.88E-07**	**24.17**	**12.02**	**33.68**	***Os05g49110***	**retrotransposon protein, putative, Ty1-copia subclass, expressed**
						*Os05g0566200*	NLI interacting factor-like phosphatase, putative, expressed
	**S1_9126931**	**7.90E-07**	**23.55**	**8.03**	**10.41**	***Os01g16160***	**retrotransposon protein, putative, unclassified, expressed**
	**S12_17888167**	**1.22E-05**	**18.03**	**5.55–9.61**	**16.62–17.19**	***Os12g29920***	**retrotransposon protein, putative, Ty3-gypsy subclass, expressed**
						*Os12g0484375*	conserved hypothetical protein
KBAC	S8_6811	5.78E-06	18.35	4.29–5.25	1.02–1.08	*Os08g01010*	hypothetical protein
LER	**S9_17873147**	**8.72E-06**	**16.69**	**4.19–6.44**	**1.00–16.07**	***Os09g0469400***	**glycogen operon protein glgX, putative, expressed**
						*Os09g0469900*	queuine tRNA-ribosyltransferase, putative, expressed
GC	S10_17333120	9.24E-06	18.39	3.91	8.82	*Os10g0469000*	leucine-rich repeat receptor protein kinase EXS precursor, putative, expressed
	**S7_24356119**	**1.07E-05**	**18.11**	**5.62**	**37.55**	***Os07g0597400***	**expressed protein**
RS	S2_22999187	1.83E-06	19.31	4.20–5.53	1.60–3.37	*Os02g0594100*	protein kinase domain containing protein, expressed
	S8_15000020	1.85E-06	19.29	4.82–6.57	1.87–4.47	*Os08g0335500*	protein kinase, putative, expressed
	S10_4535302	3.52E-06	18.15	5.64–7.50	1.80–4.21	*Os10g08370*	transposon protein, putative, CACTA, En/Spm sub-class, expressed
	S2_5050167	4.86E-06	17.58			*Os02g0191000*	disease resistance protein RPM1, putative, expressed
	S1_8845858	6.13E-06	17.17	4.33–7.14	2.70–7.73	*Os01g15720*	tobamovirus movement protein containing protein, expressed
	S6_5819355	8.84E-06	16.53	5.37–6.93	1.57–3.27	*Os06g11100*	retrotransposon protein, putative, unclassified, expressed
	S6_9155098	9.38E-06	16.43	3.59–4.59	1.45–2.80	*Os06g16080*	transposon protein, putative, CACTA, En/Spm sub-class, expressed
	S11_11627020	1.00E-05	16.32	3.82–5.12	1.69–3.79	*Os11g20120*	retrotransposon protein, putative, unclassified, expressed
						*Os11g20130*	retrotransposon protein, putative, unclassified, expressed
	S2_9683531	1.26E-05	15.92	4.53–6.49	2.11–5.46	*Os02g16970*	retrotransposon protein, putative, unclassified, expressed
PGI	**S11_5737145**	**4.88E-07**	**23.47**	**6.23–7.83**	**5.71–23.55**	***Os11g10500***	**retrotransposon protein, putative, Ty1-copia subclass, expressed**
	S11_12661155	6.93E-07	22.77	4.42–6.15	1.46–5.55	*Os11g22020*	retrotransposon protein, putative, unclassified, expressed
						*Os11g22030*	retrotransposon protein, putative, unclassified, expressed
	S1_20415780	1.99E-06	20.71	5.09–15.20	4.49–17.64	*Os01g0548000*	expressed protein
	S9_17671895	2.33E-06	20.41	4.84–14.32	4.27–16.76	*Os09g29090*	retrotransposon protein, putative, unclassified, expressed
	S7_28567910	3.61E-06	19.57	6.17–19.09	5.41–21.35	*Os07g0675200*	expressed protein
						*Os07g0675300*	expressed protein
	S8_25864590	5.29E-06	18.84	5.66–9.74	2.77–10.77	*Os08g0520300*	RNA recognition motif containing protein, putative, expressed
						*Os08g0520400*	spermidine synthase-related, putative, expressed
	S9_7528951	6.83E-06	18.35	4.00–11.40	3.51–13.73	*Os09g13060*	retrotransposon protein, putative, Ty3-gypsy subclass, expressed
	S3_21132448	8.81E-06	17.87	4.53	3.51	*Os03g38070*	retrotransposon protein, putative, unclassified, expressed
	S8_7447826	9.30E-06	17.77	4.18–4.71	1.33–5.03	*Os08g12580*	retrotransposon protein, putative, unclassified, expressed
						*Os08g12590*	retrotransposon, putative, centromere-specific

AC—Amylose content, KL—Kernel length, KB—Kernel breadth, LBR—Length/Breadth ratio, ASV—Alkali spreading value, KLAC—Kernel length after cooking, KBAC—Kernel breadth after cooking, LER—Linear elongation ratio, GC—Gel consistency, RS—Resistant starch, PGI—Predicted glycemic index, TPR—Transplanted rice and significant associations were detected with the *P* value < 0.00001 in MLM and LOD > 3 in mrMLM. Bolded MTAs shown strong association confirmed by MLM and mrMLM with high value of PVE.

To confirm the real associations, multi-locus GWAS (mrMLM) was also conducted with three models mrMLM^37^, FastmrMLM^38^ and FASTmrEMMA^39^ by adopting the critical threshold of significance for SNP-trait association was set at a LOD score of greater than 3. On comparing both MLM and mrMLM, we found that all the MTAs identified by MLM were also detected by any one method of mrMLM namely mrMLM, FastmrMLM and FASTmrEMMA with significant LOD score (Table 1, Table S2a). In mrMLM GWAS, S6_6896749 and S11_28809145 exhibited with AC with a high PVE range of 11.52 to 21.83 and 9.26 to 11.50%, S5_5361877 for KB and LBR with PVE of 1.01 to 5.29% and 1.01 to 12.26%, S6_6711302 for ASV with the PVE of 10.55 to 27.19%, S12_17888167 for KLAC with 16.62–17.19% of PVE, S9_17873147 for LER with 1.00–16.07% PVE, S7_24356119 for GC with 37.55% PVE and S11_5737145 for PGI with PVE of 5.71 to 23.55%. As per the model of mrMLM GWAS, the common MTAs identified shown higher values of PVE in the range of 5.29% (S5_5361877 for KB) to 33.68% (S5_28169331 for KLAC). The strong association MTAs identified by MLM with high PVE or R^2^ value were also confirmed by mrMLM models.

### Detection of favorable SNP alleles associated with a trait of interest

Associated MTAs of RS, PGI and nine quality traits were subjected for the identification of favorable SNP alleles (Table S2b). In this investigation, the positive effect of candidate SNP alleles that led to increase in AC, KL, L/B ratio, ASV, KLAC, LER, GC, and RS or decrease in KB, KBAC and PGI were defined as “favorable alleles”, and those that resulted in decrease of AC, KL, L/B ratio, ASV, KLAC, LER, GC, and RS or an increase in KB, KBAC and PGI were defined as “unfavorable alleles”. As a result, one MTA for each trait like AC, ASV, GC and RS had an increased phenoptypic effect whereas KB, KBAC and PGI revelaed decreased effect in their phenotype are designated as favorable alleles. S6_6896749 possessing ‘G’ allele had strong phenotypic effect (0.88%) on AC, S5_5361877 possessing ‘A’ allele with 0.18 of L/B ratio, S6_6711302 with ‘T’ allele had 1.96 phenotypic effects on ASV, S6_5819355 with ‘A’ allele had 1.74% increase in phenotypic effect in RS and S8_7447826 with ‘A’ allele had resulted in decrease of − 2.91 in PGI (Table S2b). Findings of this study indicated that the favorable SNP significantly increased or decreased the phenotypic effect of the trait in the genotypes they were present. Identification of superior haplotypes of these mined favorable alleles would be beneficial to develop elite entries with preferred grain quality and lower GI value.

### Haplotype analysis of identified candidate loci

All the MTAs associated with the quality-related traits were subjected to the identification of candidate loci using RAP database (Table S2b). Out of the 41 MTAs detected in this investigation, we found 27 MTAs were within the candidate loci and 14 MTAs were found to be flanked by two candidate loci. Of these, nine loci for PGI, nine loci for AC, nine loci for RS, four loci each for KB, KLAC, two loci for GC, one loci each for KL, LBR, ASV, LER and KBAC were identified in this study.

The identified candidate loci were subjected to haplotype analysis by rice SNP seek database to estimate the number of haplotypes present in the sub-set of 3 K-RGP for all the investigated quality-related traits and to use superior haplotypes to breed quality rice varieties. Haplotype analysis reported a minimum of one haplotype to the maximum of 15 haplotypes for the identified loci. The loci *Os08g0276000, Os02g0135800* and *Os10g09430* for AC, *Os05g09510* for KB and LBR, *Os05g0566200* and *Os12g0484375* for KLAC, *Os09g0469400* for LER, *Os02g0594100*, *Os02g0191000*, *Os11g20120* and *Os11g20130* for RS and *Os08g0520400* for PGI had registered one haplotype, respectively. The loci namely, *Os06g18710* and *Os06g18720* for AC, *Os05g0187500* for KB and LBR, *Os06g12380* for ASV, *Os08g01010* for KBAC, *Os06g16080* and *Os02g16970* for RS and *Os07g0675300* for PGI were found to be recorded 15 haplotypes explaining maximum diversity. On the other hand, 14 loci had 2 haplotypes, 7 loci had 3 haplotypes, 4 loci had 4 haplotypes, 3 loci had 5, 1 locus had 8, 10 and 14 haplotypes, respectively (Table S2b).

### Haplotype frequency of identified causative genes

The major loci *Os06g0232700* associated with AC had five haplotypes where H5 representing the higher frequency of 57.33%. The other loci on chromosome 6, *Os06g18710* representing maximum haplotype diversity of 15 haplotypes where H4 recorded a higher frequency of 52.66% (Table S3). The candidate SNP for KL centered between two loci namely *Os11g0706100* and *Os11g0706500* exhibited a higher frequency of H5 with 58.66% and H3 with 42.66%, respectively. S5_5361877 is the major allele for KB and LBR falls within candidate genes namely *Os05g09510* and *Os05g0187500*, where *Os05g0187500* possessing 15 haplotypes with the higher haplotype frequency of 48.66% in H14. The trait ASV had an association with S6_6711302 within two loci, in which *Os06g12380* had 15 haplotypes where H5 recorded the maximum frequency of 41.33%.

### *Haplo-Pheno* analysis for the identification of superior haplotypes

**Figure 4:**
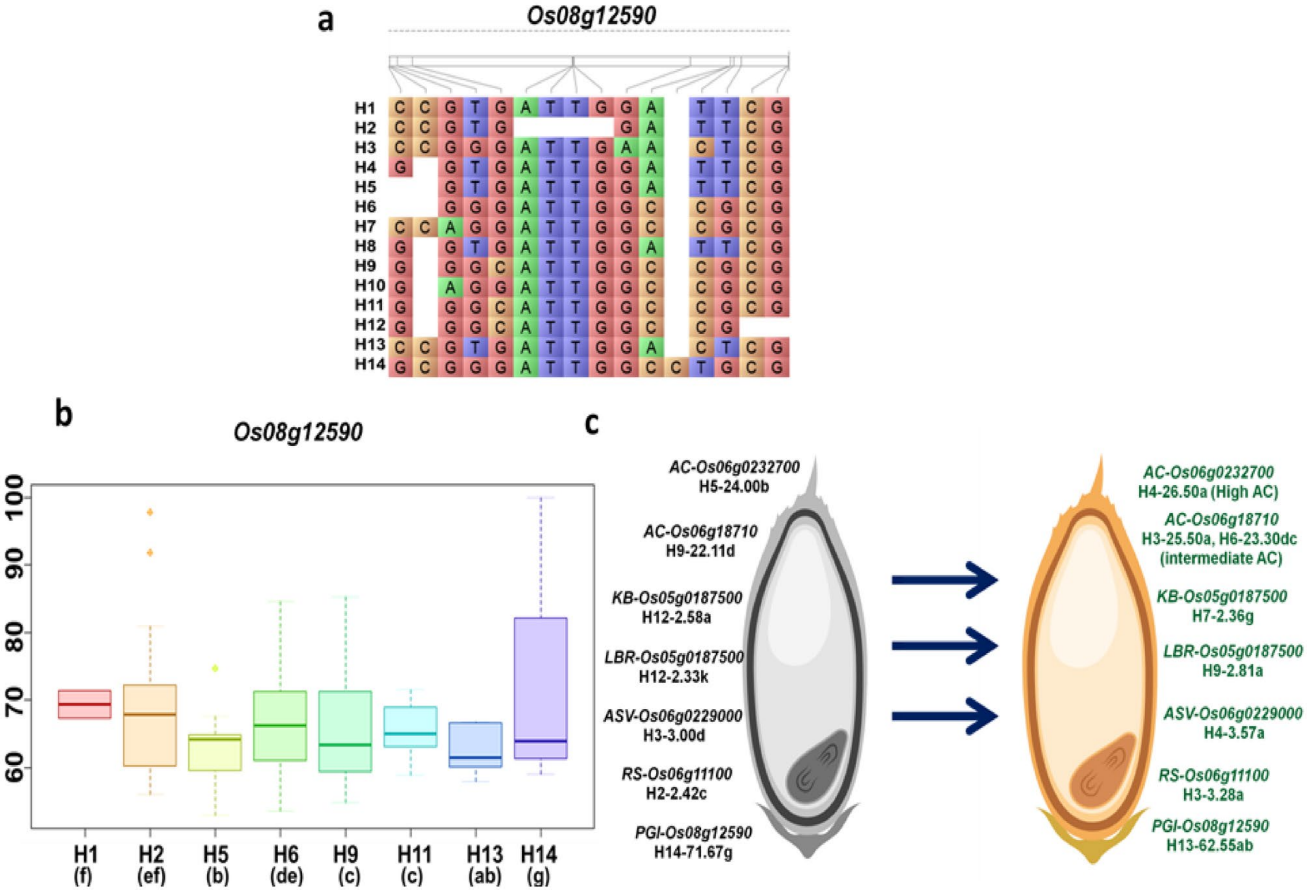
(**a**) Haplotype variations observed for *Os08g12590* associated with predicted glycemic index in TPR situation, (**b**) Boxplot shown haplotype diversity and variations exists between haplotypes of *Os08g12590* linked with PGI with the significance level of *p* value < 0.05 and different alphabets denotes significant differences between haplotypes, (**c**) Assembly of superior haplotypes (Haplotype based breeding) of RS, PGI and other important quality traits with favorable allelic effect to design diabetic free healthy rice with preferred grain quality and genetic gains.

For a better understanding of phenotypic performance of the accessions carriying specific haplotype, trait mean was worked out for each haplotype separately. Here, candidate loci possessing more than 2 haplotypes were statistically analyzed by Duncan’s test to identify the superiority of the haplotype for the identified MTAs associated with RS, PGI and other quality traits (Table 2). Once MTAs with favorable alleles identified, it was subjected to haplotype analysis with the help IRRI SNP seek database. The candidate loci associated with AC on chromosome 6 namely *Os06g0232700* (H4- 26.50% for high amylose; H5-24.00% for intermediate amylose), *Os06g18710* (H3-25.50%; H6-23.30% for intermediate amylose**)** and *Os06g18720* (H4-26.58% for high amylose; H14-23.36% for intermediate amylose) had shown positive allelic effect with the superior haplotype of increasing in amylose content. For RS and PGI, the candidate loci *Os06g11100* (H4-3.28% for high RS; H2-2.42% for intermediate RS) and *Os08g12590* (H13-62.52 as intermediate PGI) (Fig. 4) possesses positive and negative allelic effect of 1.74 and -2.91, respectively. For grain size (LBR), H9-2.81 of *Os05g0187500* had the positive allelic effect of 0.18. For ASV and GC, H1-3.43 and H1-56.06 as the desirable haplotype for the candidates of *Os06g0229000* and *Os10g046900* (Table 2).

**Table 2:** Haplo-pheno analysis of favourable candidate loci for grain quality traits, RS content and predicted glycemic index in rice.

Trait	Gene ID (RAP)	Allelic effect	Gene function	Average performance of superior haplotype	Average performance of other haplotype	Lines with superior haplotype	Disrtibution of Superior Haplotype		
							*Indica*	*Aus*	*Admixture*
AC	*Os06g0232700*	0.88	transposon protein, putative, unclassified, expressed	H4- 26.50^**a**^	H1-24.25^**b**^, H2-24.44^**b**^, H3-24.03^**b**^, H5-24.00^**b**^	4	4	0	0
	*Os06g18710*	0.28	retrotransposon protein, putative, unclassified	H3-25.50^**a**^	H1-24.50^**b**^, H4-24.12^**b**^, H6-23.30^**c**^, H7-24.28^**b**^, H9-22.11^**d**^ and H15-24.37^**b**^	2	1	1	0
	*Os06g18720*	0.28	retrotransposon protein, putative, Ty3-gypsy subclass, expressed	H4-26.58^**a**^	H1-20.00^** g**^, H2-24.45^**d**^, H3-25.00^**c**^, H5-23.80^**e**^, H6-24.64^**d**^, H7-24.9^** cd**^, H8-25.75^**b**^, H10-23.11f., H12-19.5^** h**^, H13-24.75^** cd**^, H14-23.36^**ef**^	3	3	0	0
KB	*Os05g0187500*	-0.1	IQ calmodulin-binding motif family protein, expressed	H7-2.37^**a**^	H1-2.4^**b**^, H2-2.42^**c**^, H3-2.55^**b**^, H4-2.4^**b**^, H5-2.56f., H6-2.46^**de**^, H9-2.4^**b**^, H11-2.47^**e**^, H12-2.58^** g**^, H13-2.45^**d**^, H14-2.46^**de**^, H15-2.46^**e**^	6	4	2	0
LBR	*Os05g0187500*	0.18	IQ calmodulin-binding motif family protein, expressed	H9-2.81^**a**^	H1-2.71^**b**^, H2-2.69^**c**^, H3-2.14^**i**^, H4-2.66^**de**^, H5.2.38^**j**^, H6-2.68^** cd**^, H7-2.66^**e**^, H11.2.59^** g**^, H12-2.33^** k**^, H13-2.61f., H14-2.57^** h**^, H15-2.51^**i**^	73	72	0	1
ASV	*Os06g0229000*	1.96	OsFtsH6 FtsH protease, homologue of AtFtsH6, expressed	H1-3.43^**c**^	H2-3.47^**b**^, H3-3.00^**d**^, H4-3.57^**a**^	9	8	1	0
	*Os06g12380*	1.96	expressed protein	H11-2.75^**c**^	H5-4.48^** h**^, H6-2.92^**d**^, H8-3.67^** g**^, H9-3.25f., H10-3.00^**e**^, H13-2.58^**b**^, H15-2.60^**b**^	8	8	0	0
KBAC	*Os08g01010*	-1.16	hypothetical protein	H11-3.17^**a**^	H1-3.40f., H3-3.52^** g**^, H4-3.31^**d**^, H6-3.18^**b**^, H10-3.29^**c**^, H13-3.35^**e**^, H14-3.53^** g**^, H15-3.60^** h**^	8	8	0	0
GC	*Os10g0469000*	5.01	leucine-rich repeat receptor protein kinase EXS precursor, putative, expressed	H1-56.06^**a**^	H2-47.73^**b**^	6	5	1	0
RS	*Os06g11100*	1.74	retrotransposon protein, putative, unclassified, expressed	H4-3.28^**a**^	H1-3.06^**a**^, H2-2.42^**c**^, H3-2.85^**ab**^	48	48	0	0
PGI	*Os08g12590*	-2.91	retrotransposon, putative, centromere-specific	H13-62.52^**ab**^	H1-69.34f., H2-67.64^**ef**^, H5-63.36^**b**^, H6-66.68^**de**^, H9-65.41^**c**^, H11-65.53^**c**^, H14-71.67^** g**^	5	5	0	0

AC—Amylose content, KL—Kernel length, KB—Kernel breadth, LBR—Length/Breadth ratio, ASV—Alkali spreading value, KLAC—Kernel length after cooking, KBAC—Kernel breadth after cooking, LER—Linear elongation ratio, GC—Gel consistency, RS—Resistant starch, PGI—Predicted glycemic index, TPR—Transplanted rice.

For KL, *Os11g0706100* had H8 (6.71 mm) and H7 (5.67 mm), *Os11g0706500* had H2 (6.41 mm) as a desirable haplotype for development of long and medium slender grain rice varieties . *Os05g0187500* and *Os03g35870* were associated with KB had H7 (2.37 mm) and H1 (2.42 mm) as superior haplotypes for the corresponding loci. For LBR, *Os05g0187500* had H9 as desirable haplotype having genotypes recorded higher length-breadth ratio (2.89) possessing slender grain rice lines. *Os06g0229000* and *Os06g12380* associated with ASV showed H4 (3.67 of mean score) and H5 (4.48 of mean score) as anticipated one since intermediate ASV is desirable to breed good quality rice varieties. The other loci for RS *Os06g16080* registered H11 (5.37%) for high and H7 (2.95%) as desirable haplotypes for intermediate RS content, respectively. Other loci, *Os02g16970* linked with RS reported H14 (4.49%) as a superior haplotype for improving RS content (Table S4).

## Discussion

Rice, one of the essential staple food crop species is consumed by half of the global population especially in Asian continents. The ever-increasing population of the world demands more than 50% additional rice production to be attained by 2030 to meet future needs^40^. Dissecting the complexity of grain quality is possible by means of genome wide association mapping using a diverse set of population with a higher precision approach to map the causative alleles than the bi-parental mapping population since GWAS population consist higher evolutionary recombination events in their genomes^41^. During the past three decades, tremendous efforts have been put in by rice breeders and geneticists to detect the causative QTLs/genes responsible for quality improvement in rice and many researchers came up with remarkable results. Grain quality includes cooking and eating quality of rice grains and these are complex traits governed by multiple genes with various gene expression and regulatory network pathways and several QTLs/genes associated with grain quality were identified and cloned in rice^42^. Starch biosynthesis in rice grains during grain formation and development is one of the critical factors in improving the quality aspects of rice varieties and regulation of this complex metabolic pathway controlled by a network of various genes and gene combinations are poorly understood. *Waxy* gene (*GBSSI*) plays a key role in amylose biosynthesis and the formation of long-chain amylopectin^43^. Another gene *Starch synthase IIa* (*SSIIa*) modifies amylopectin structure and improving grain quality^44,45^. Likewise, several other genes namely *SSI*, *SSIIIa*, *isoamylase1* (*ISA1*), *pullulanase* (*PUL*), branching enzyme (*BEI* and *BEIIb*)^46–50^, have also been reported to control starch structure formation and modifies the physicochemical properties of rice grains. Therefore, amylopectin content and structure alteration have a significant impression on the modification of starch granules and affect cooking properties in rice.

In recent times, non-infectious diseases cause several health-related issues like cardiovascular problems, obesity and diabetics due to the high consumption of calories and improper physical exercises. Recent study reported that high amylose starch-rich RS decreases the glycemic value of starch, insulin response in human metabolism and reduce the risk of type II diabetes mellitus and other health-related issues^51^. Currently, breeders and geneticists are making efforts to develop high RS rice varieties. Cultivars rich in amylose content of rice, maize and barley developed by either mutational breeding or biotechnological approaches have been found to possess high RS content^52–54^.

In the present investigation, diverse panel of rice accessions (3 K-RGP sub-set) were not only phenotyped for grain quality-related traits but also phenotyped for the most important health/well-being related traits such as RS and PGI to map candidate SNPs, functional annotations and to unravel the haplotype diversity of identified candidate loci across the 3 K-RGP. Then, the *haplo-pheno* analysis was carried out to ascertain superior or appropriate haplotype for the improvement of grain quality in rice breeding programs.

RS, a valuable trait was found to have a significant positive correlation with amylose content and was negatively correlated with glycemic index as suggested by several previous reports and thus a suitable parameter for rice consumption to improve the health status of those who are suffering from diabetics, colon cancer, and obesity^7^. We identified nine strong MTAs for RS with significant PVE on chromosomes 1, 2, 6, 8 and 11 (Table 1). Primarily, previous reports found that *sbe3-rs* on chromosome 2^55,56^ and *SSIIIa* (*Os08g0191433*) on chromosome 8^45^ involved in the production of RS content and it may be associated with expression of *Waxy* gene as well. In our study, we report two MTAs in proximity to the candidate regions mentioned above of chromosome 2 (*Os02g0594100*) and chromosome 8 (*Os08g0335500*) with not much significant haplotype diversity but significant variations in the trait value was observed among the accessions^57^. We also identified a locus on chromosome 6 (*Os06g11100*) near (1 Mb away) to the position of *starch synthase IIa* (*SSIIa*: *Os06g0229800,* 6,748,398–6,753,302) gene, significantly affecting GC, degree of starch crystallinity by increasing the fractional amount of A chains in amylopectin^8,44,58^. All the identified MTAs and their corresponding candidate loci exhibit a significant number of variable haplotypes for the traits studied. In recent past, the discovery of mutation/variants each in *GBSSI*, *SSI*, *SSIIa*, and *SSIIIa* with a potential to increase RS content and hydrolysis index in rice has been reported^59,60^. For PGI, we identified a total of 9 associations on chromosome 1 (*Os01g0548000*), 3 (*Os03g38070*), 7 (*Os07g0675200*), 8 (*Os08g12580* and *Os08g0520300*), 9 (*Os09g13060* and *Os09g29090*) and 11 (*Os11g10500* and *Os11g22020*) (Table 1). Similar kind of results were reported^9^ and we also found significant MTAs influencing PGI in close proximity on chromosome 1 (snp_01_30302075 and snp_01_36980087), Chr 3 (snp_03_22422723), Chr 9 (snp_09_21456056 and snp_09_21523496) and Chr 11 (snp_11_334055 and snp_11_28758733). Similar is the case for GC; our results match with* “qAC7”* associated with RM8261 in the physical position Chr7: 25,866,581^61^. Here, we identified a candidate *Os07g0597400* on chromosome 7 associated with GC. A QTL *qRS7-2* on chromosome 7 associated with RS content between RM3404 (chr7:20,105,832) and RM478 (chr7:25,949,521) was identified and reported^36^.

Over several decades, quality traits like AC, GC, ASV, LER, etc., of rice grains have been extensively studied and it has been reported that AC is governed by waxy gene^62^, by several other loci^63^ and even by unidentified non-waxy genes^64^. Our study reported nine associations for AC (Fig. S1) and the major SNP S6_6896749 located with the candidate loci of *Os06g0232700* on chromosome 6 found adjacent to *ALK* gene (6,726,252)^65–67^. Several other loci were identified on Chromosome 2, *Os02g0135800* encoding for WD domain, G-beta repeat domain-containing protein, expressed and *Os02g05180* (2,474,176) encrypting for retrotransposon protein, putative Ty3-gypsy subclass. *Starch branching enzyme 3* (*SBEIIb*- *Os02g32660*) is essential for amylopectin synthesis in the endosperm^68,69^. Several reports found that high amylose rice and maize reveals in amylose extender (ae) phenotype due to the inactivation of *SBEIIb*^46^. These results clearly confirmed that a network of genes are involved in the biosynthesis of amylose. Our association analysis study provides a stronger picture of how genetic network of starch synthesizing genes involved in cooking and eating quality improvement in rice. We also identified an association on chromosome 11, S11_28809145 on *Os11g0704000* with the function of selT/selW/selH selenoprotein domain-containing protein, expressed slightly closer to *Os11g31330*^70^.

Kernel length, kernel breadth, and LBR are the important traits to assess the grain size and dimensions for selecting appropriate breeding lines for their consumer’s preference and market acceptability. Medium slender rice is more preferable than bold rice varieties. For kernel length, an MTA was identified on chromosome 11 (S11_28909306) with 9 haplotypes. For kernel breadth, one MTA was observed on chromosome 5, two on chromosome 3 and one on chromosome 2. LBR determines the grain size and is controlled by a complex network of genes and also affected by environmental factors. Our study reported similar findings of^13^, who reported quite a lot of QTLs for grain length, grain width and grain length–width ratio on chromosome 3, 5 and 7 by utilizing 3 k panel sub-set. To date, several hundred QTLs associated with grain size have been identified and functionally characterized viz., *GS3, GW2, qSW5/GW5, GS5 and qGL3/qGL3.1*^14,16,17,19,71–74^. Among the above said QTLs *GS3* and *qSW5* showed consistent and sturdy effect on grain size variations reported in our investigation also ^71,75–77^. For instance, the locus *Os05g0187500* identified having large haplotype diversity responsible for the putative function of IQ calmodulin-binding motif family protein exhibits peak association close to the SNP position of S5_5361877 reported for KB and LBR^27,31,41,78^.

ASV is an indicator for gelatinization temperature, we identified a strong and peak association on chromosome 6 at the position of S6_6711302 (between *Os06g0229000* and *Os06g12380*) with worthy haplotype diversity in the range of 4 to 15 haplotypes, respectively. The same QTL on chromosome 6 at the position of 6,726,252 associated with *ALK* gene was reported ^65–67,78^. A study conducted with 258 accessions of 3 k panel also reported the same genic location (S6_6752888) for GT and a candidate of *ALK* gene encoding *SSIIa* affecting the chain-length distribution of amylopectin causing alkali disintegration of rice grains^13,65,79^. For gel consistency, we identified two candidates on chromosome 7 (*Os07g0597400*) and 10 (*Os10g0469000*) with minimum haplotype variations of 8 and 2. Previously, a *qGC6* was reported between close proximity to *WAXY* gene^13^. More importantly, a new novel association for GC is identified on chromosome 7 had near vicinity and it was not previously reported. Linear elongation ratio is an important cooking quality trait and we identified an associations for LER on chromosomes 9. A QTL for LER was reported on chromosome 4 with the marker interval of C933 to C946 using RIL population of *indica* x *indica* hybrids^80^.

On a whole, amylose and amylopectin production is a complex phenomenon governed by several networks of genes and their coordinating expression during grain filling and seed maturation affecting the grain textural properties and even prevailing environmental conditions also. Noteworthy, it is not an easy breeding protocol to improve the quality aspects of rice varieties by selecting a notable gene that may be either superior or inferior for a specific trait and introgression by markers assisted pyramiding and selection. In this, we identified several superior haplotypes by studying haplotype diversity and linking haplotype variations with the trait value. Assembling superior haplotype combinations for most of the grain quality traits viz., AC, ASV, GC, LBR and RS coupled with GI in to one background to maximize the textural properties of grains, yield and most of morpho-agronomic traits to develop tailor-made new generation rice with enhanced genetic gain compared to available popular mega rice varieties^81,82^. For example, AC (S6_10612428) had maximum haplotype diversity with the superior haplotype of H3 (*Os06g18710* – 25.50%) possessing high amylose class, LBR (*Os05g0187500*) owns H9 (2.81) as superior one among 15 haplotypes, RS (*Os06g11100*) holds H4 (3.28%) as topmost one from all the 4 haplotypes.

In this investigation we also identified better donors for RS, PGI and other quality-related traits which can be utilized in the *haplotype-based breeding* program to develop elite lines with low glycemic index value with desirable quality traits by assembling superior haplotypes suited for different situations (Table S5). Our results were compared with previous reports of glycemic index, we found UQUIHUA::IRGC 117,037–1 had registered intermediate GI of 66.88 with the RS content of 3.23% whereas^9^ reported as low GI (< 55). Apart from this we also selected different classes of glycemic index lines from the 3 K sub-panel grown in 2019WS and different quality-related traits were phenotyped and result showed that four lines namely BAIANG 6::IRGC 6129–1 from Indonesia, MAKRO::IRGC 74,763–1 from India, AUS 329::IRGC 29,116–1 from Bangladesh and KOTTEYARAN::IRGC 47,383–1 from Srilanka possesses medium slender grains with good elongation ratio and medium to soft gel consistency after cooking (Table S6). The line MAKRO::IRGC 74,763–1 from India having intermediate RS content and low PGI of 52.91.

But when considering ASV for GT, the identified entries for the low glycemic index showed the score of 1 to 2 which represents high GT requires more water, cooking time and poor in texture not suitable for cooking and eating (Table S6). Amylose content alone does not describe the cooking and eating quality of rice grains, as varieties with the same range of AC possess variable variations in the cooking and eating quality^55^. In our study, intermediate ASV of 4 to 5 score possesses high PGI values. Superior haplotypes associated with the low glycemic index, intermediate ASV and soft GC in the preferred combinations can be utilized for the development of low glycemic rice varieties with desirable grain quality. Hence, the approach of *haplotype-based breeding* is anticipated to assist in the development of -premium quality rice varieties with low GI to meet the increasing demands of the rice consuming population.

## Conclusion

Significant variations were observed for the grain quality traits, especially for AC, ASV, GC and RS. We captured several novel and significant associations of SNPs for the target traits and studied haplotype differences of identified candidate genes. Newly identified candidate genes might be useful for futher functional characterization and pathway elucidation for grain quality traits in rice. Here we proposed an exclusive strategy on use of superior haplotype-possessing elite donors and incorporating superior/appropriate haplotypes for the majority of quality traits in a genetic background as a prominent way to develop high yielding and quality rich rice varieties suitable for the consumption of diabetics, obese population and as preferred by consumer’s needs and demands.

## Material and methods

### Genetic material and field trial

The genetic material used in this study comprised of 150 accessions having different duration groups of 3 K RGP evaluated under transplanted situation (TPR) during dry season 2017 (DS2017) and Wet season 2018 (WS2018) (Table S7). These accessions were evaluated for RS, PGI and 9 quality parameters including physico-chemical properties, cooking and eating properties. The analysis was carried out at the rice quality lab at IRRI SA Hub, ICRISAT campus. The agronomic management of the transplanted experiments including was carried as per the procedure detailed^83^.

### Phenotyping of physicochemical parameters and cooking and eating quality of rice

Traits namely, KL, KB and LBR were measured with 10 kernels using Standard Evaluation System of Rice (SES)^84^ and the mean was calculated. Amylose content of the grains was estimated by the rapid protocol of cut grain dip method^85^. For cooking-related parameters, 20 kernels from each entry were soaked in 5 ml of distilled water in 15 ml test tubes for 20 min and cooked in a boiling water bath at 100 °C intended for the time period of 8 min to determine the KLAC, KBAC and LER. Gel consistency was determined by the method formulated by^86^, where known amount of rice flour was placed in the culture tubes and wetted with 0.2 ml 95% ethanol containing 0.03% thymol blue and 2 ml 0.2 N KOH added and mixed with a Vortex Genie mixer set at specified speed. Tubes were covered with glass marbles and heated in a vigorously boiling water bath for 8 min. Then, the tubes were removed from the water bath and kept at room temperature for 5 min, cooled in an ice-water bath for 20 min, and laid flat on a laboratory table and length of the blue-colored gel was measured in millimeters. The method separated different classes of GC into soft (61–100 mm), medium (41–60 mm) and hard gel consistency (25–40 mm) based on SES, 2013. GT was estimated based on alkali spreading value (ASV) of milled rice by^87^. Six whole-grain, milled rice samples were placed in duplicate Petri plates containing 10 ml of 1.7% KOH. The Petri plates were covered and incubated for 23 h at 30 °C. The appearance and disintegration of grains were visually observed after incubation based on the scale given by SES, 2013.

### Phenotyping for RS and predicted GI

#### Measurement of RS

RS content of the rice samples was determined by using a resistant starch assay kit (K-RSTAR, Megazyme, Irishtown, Ireland) by ten-fold downscaling of sample and reagents with slight modifications^88^. Fine powder of polished rice flour (10 mg) was taken in 2 ml eppendorf tube and it was digested with 400 μl of enzymatic mixture-containing 10 mg/ml of pancreatic α-amylase and 3.0U/ml of amyloglucosidase (AMG) and the mixture was incubated at 37^0^C for 16 h with continuous shaking (200 strokes/minute). After incubation, 400 μl of 99% ethanol was added to the mixture and vortexed to stop the reaction. Then, the samples were centrifuged for 10 min at 12,000 rpm and the supernatant was collected and the pellets were washed repeatedly with 200 μl (vortexed) and 600 μl of 50% ethanol. Washing with 50% ethanol was made twice to remove all the non-resistant starch in the samples and supernatants were pooled for the measurement of non-resistant starch. Then, the pellets were air-dried for 30 min to remove moisture content in the residue. Around, 200 μl of 2 M KOH was added to the residue and mixed properly to avoid the formation of clumps and incubated at 5^0^C for 1 h in a shaker (200 strokes/minute). Then, 800 μl of 1.2 M sodium acetate buffer and 10 μl of AMG (3,300 U/ml), vortexed and tubes were placed in a water bath for 30 min at 50^0^C with intermittent mixing with the help of vortex mixer for every 10 min. After incubation, samples were cooled and centrifuged at 12,000 rpm for 10 min. Then, 100 μl of sample aliquot was taken in a fresh test tube (15 ml), 3 ml of GOPOD reagent (D-Glucose assay kit, Megazyme, Irishtown, Ireland) and incubated in a water bath at 50^0^C for 30 min. The absorbance was determined with the help of UV-1800 (Shimadzu Corporation, Japan) at 510 nm against a blank containing buffer and GOPOD reagent.

#### Estimation of predicted GI (PGI)

The glycemic index was determined by the protocol given by^10^ with little modifications. Fine powder of polished rice flour (50 mg) was cooked in 5 ml of water for 30 min and 10 ml of HCl-KCl buffer (pH = 1.5) was added to the sample. Then, 0.2 ml of a solution containing 1 g of pepsin in 10 ml of HCl-KCl buffer was added to each sample and incubated at 40 °C for 1 h in a shaking water bath. After incubation, the volume was adjusted to 25 ml with Tris maleate buffer (pH = 6.9). Then, 5 ml of α–amylase (2.6 units) in Tris maleate buffer (pH 6.9) was added and samples were incubated at 37 °C in a shaking water bath. After incubation, 1 ml of the sample aliquot was collected serially at intervals of 30 min for 3hrs (30, 60, 90, 120 and 180 min). The enzyme activity in the aliquot was inactivated by heating at 100 °C for 5 min and refrigerated until the end of the incubation period. 3 ml of 0.4 M Sodium acetate buffer (pH 4.75) and 60 µl amyloglucosidase was added to hydrolyze the digested starch to glucose. The samples were incubated at 60 °C for 45mins in a water bath and glucose content in each aliquot was estimated using the GOPOD kit (D-Glucose assay kit, Megazyme, Irishtown, Ireland). Glucose was converted into starch by multiplying with 0.9. Kinetics of starch digestion was estimated by non-linear first-order equation; C = Cα (1-e^-kt^). Where, C = Concentration of starch hydrolyzed at the time (t) , Cα = equilibrium concentration (i.e., % of starch hydrolyzed after 180 min which is the glucose content after 120 min divided by the total starch) and k = kinetic constant. The area under the hydrolysis curve (AUC) was calculated using the equation; AUC = Cα _(tf-t0)_—Cα/k (1-e-^k (tf – to)^). Where, C∞ corresponds to the concentration at equilibrium (t_180_), t_f_ is the final time (180 min), t_0_ is the initial time (0 min) and k is the kinetic constant. A hydrolysis index (HI) was calculated by comparison with the AUC of a reference food (white bread). The predicted glycemic index (PGI) was estimated using the formula PGI = 39.7 + 0.548 (HI).

### GWAS, Haplotype analysis and *Haplo-Pheno* analysis

Mean data was used for GWAS analyses using approximately more than 500 K SNP data. The analysis was carried out by using GAPIT (MLM model) (https://CRAN.R-project.org/), R based approach considering both kinship values (k-values) and population structure (Q-matrix)^89^. Of the several methods suggested to correct false positive in association analysis even keeping stringent p-value benchmark, the most stringent correction method called “Bonferroni Correction” was used in the present analysis.The bonferroni threshold calculated by the formula 1/m where ‘m’ is the number of test performed which resulted in –log_10_ (1/m) = 5.69. As it is too conservative and suggestive value of *p* = 0.00001 (− log_10_ (*p*) > 5)^31,90^ was used for the identification of peak associations (MTAs) with the targeted grain quality traits. Multi-locus GWAS (mrMLM) was also conducted to validate the MTAs identified by single locus model of MLM by adopting the significant LOD score of 3^91^. The phenotypic allele effect (ai) was determined by the formula given by^29^ and the favorable alleles of each trait were subsequently identified according to the breeding objective. Strongly associated SNPs with the targeted traits were utilized to find the causative QTLs/genes by the RAP database. In-built tool of SNP seek database^89^ was utilized to conduct haplotype analysis for entire candidate locus, by employing default parameters with Calinski criteria for k-group determination. Nipponbare was used as the reference genome. All of the 150 lines belonging to 8 sub-populations namely, aro, aus, admix, ind1A, ind1B, ind2, ind3, and indx was considered for the haplotype analysis. We utilized the ‘3kfiltered’ SNP set present in the SNP seek database for the entire analysis. The filtered was obtained from the Base SNP set by applying the following filtering criteria: (1) alternative allele frequency at least 0.01, (2) proportion of missing calls per SNP at most 0.2^92^ (http://snp-seek.irri.org/_download.zul) and this SNP set was already available in the SNP seek database which was directly utilized in this study. Haplotype analysis for candidate locus have been carried out considering only the nonsynonymous SNPs and indels in the exon region that results in amino-acid change. The information regarding haplotype and their diversity was obtained from the SNP-seek database (http://snp-seek.irri.org/_download.zul) to detect the superior haplotype by categorizing haplotypes by using phenotyping data of concerned genotype trait means for the associated genes.

### Statistical analysis

Mean, range and standard deviation were calculated with the help of a standard excel program. Duncan’s multiple range tests were carried out using XL Stat 2019. Correlation analysis among all the component traits related to quality was conducted by SPSS ver. 20. Boxplot and correlogram were constructed with the help of R-package (https://CRAN.R-project.org/). Significant difference among the haplotypes (only the haplotypes validated in at least two lines were considered) was studied with the help of F-statistic and Duncan’s test with the significance level of *p* < 0.05.

## Supplementary Information

Supplementary Information 1.

Supplementary Information 2.

Supplementary Information 3.

## Abbreviations

AC: Amylose content
KL: Kernel length
KB: Kernel breadth
LBR: Length/ Breadth ratio
ASV: Alkali spreading value
KLAC: Kernel length after cooking
KBAC: Kernel breadth after cooking
LER: Linear elongation ratio
GC: Gel consistency
RS: Resistant starch
PGI: Predicted glycemic index
TPR: Transplanted rice
MTAs: Marker trait associations
GWAS: Genome wide association studies
