# Supplementary material for: Superior haplotypes towards development of low glycemic index rice with preferred grain and cooking quality
Source: Sci Rep. 2021 May 12;11:10082. doi: 10.1038/s41598-021-87964-8 (PMC8115083; doi:10.1038/s41598-021-87964-8)
Supplement: Supplementary file 3 — Supplementary Information 3. [file 41598_2021_87964_MOESM3_ESM.docx]

**Superior haplotypes towards development of low glycemic index rice with preferred grain and cooking quality**

*Ramchander Selvaraj*^1^**^†^***, Arun Kumar Singh*^1^**^†^***, Vikas Kumar Singh*^1^**^†^***, Ragavendran Abbai*^3^*, Sonali Vijay Habde^2^, Uma Maheshwar Singh*^2^ *and Arvind Kumar*^1^

**Running title:**

*Superior haplotypes for healthier rice*

**Arvind Kumar^1*^**

^1^IRRI South Asia Hub (IRRI-SAH), ICRISAT Campus, Patancheru, Hyderabad, India

^2^International Rice Research Institute (IRRI), South-Asia Regional Centre (SARC), Varanasi, India

^3^Leibniz Institute of Plant Genetics and Crop Plant Research (IPK), Gatersleben, Germany

^*^Author for Correspondence

Arvind Kumar

IRRI South Asia Hub (IRRI-SAH), ICRISAT Campus, Patancheru, Hyderabad, India

Hyderabad, Telangana

India.

Email: [arvind.kumar@cgiar.org](mailto:arvind.kumar@cgiar.org)


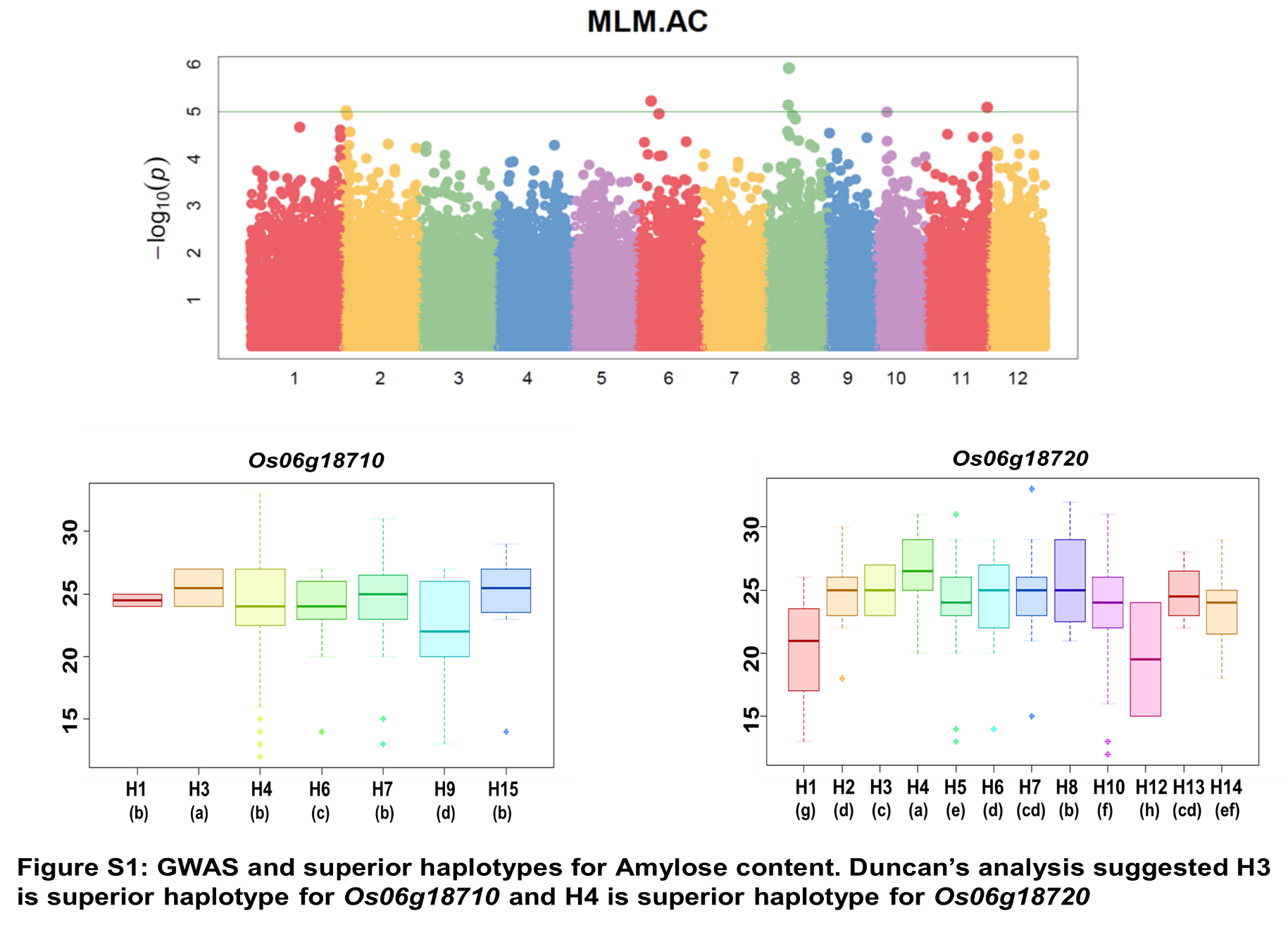


**Figure S1:** GWAS and superior haplotypes for Amylose content. Duncan’s analysis suggested H3 is superior haplotype for Os06g18710 and H4 is superior haplotype for *Os06g18720*

**
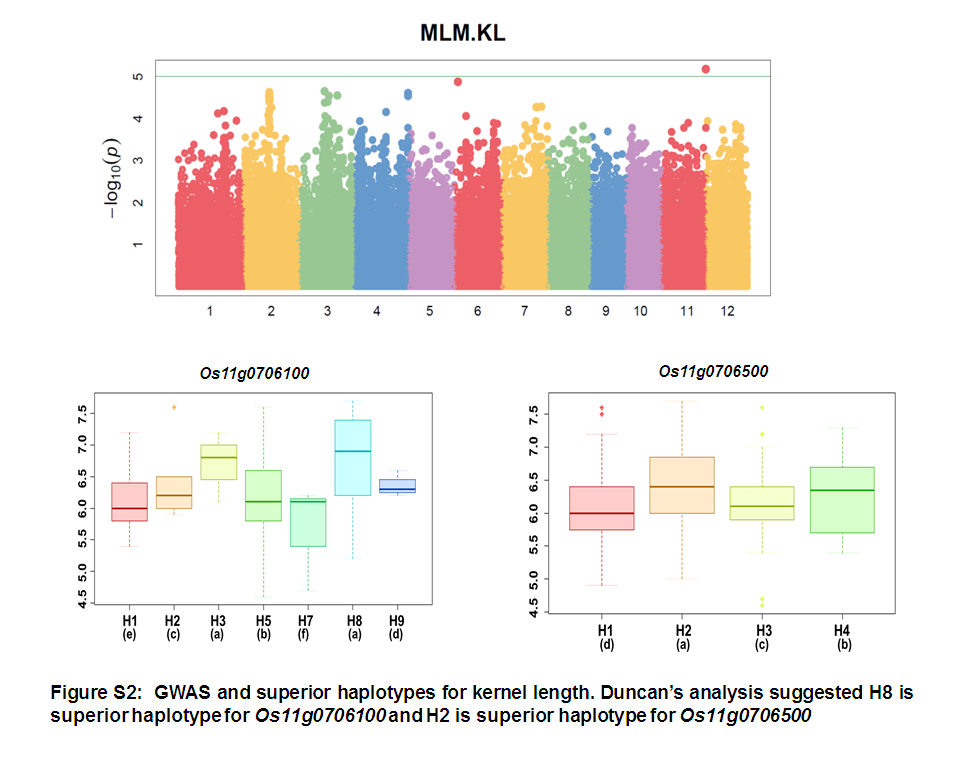
**

**Figure S2:** GWAS and superior haplotypes for kernel length. Duncan’s analysis suggested H8 is superior haplotype for *Os11g0706100* and H2 is superior haplotype for *Os11g0706500*


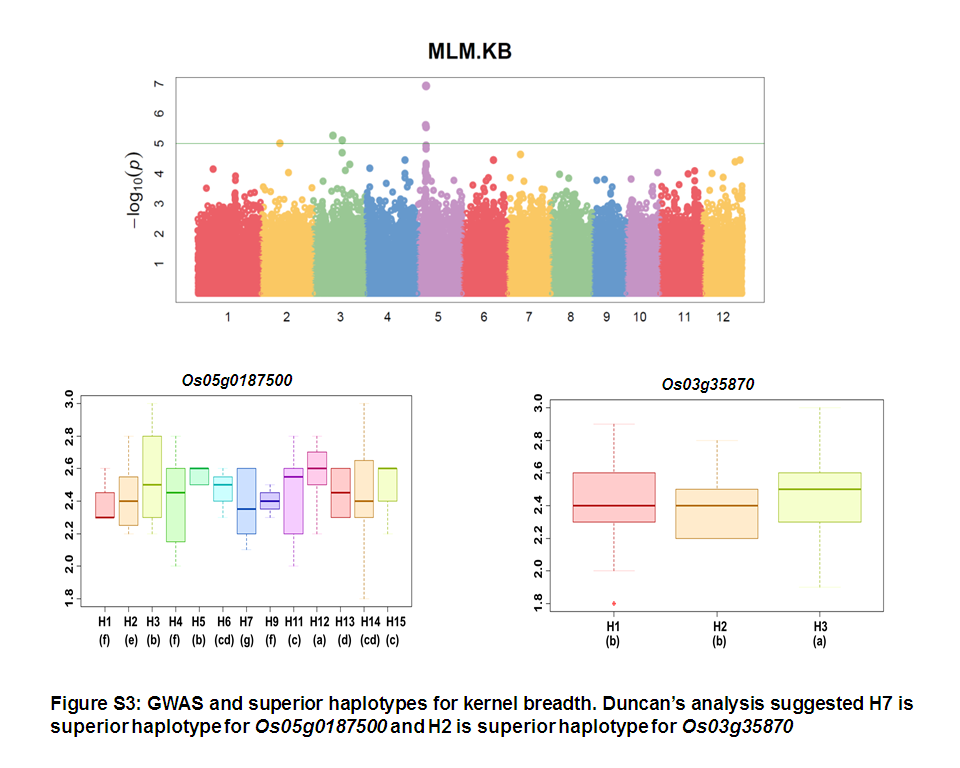


**Figure S3:** GWAS and superior haplotypes for kernel breadth. Duncan’s analysis suggested H7 is superior haplotype for *Os05g0187500* and H2 is superior haplotype for *Os03g35870*


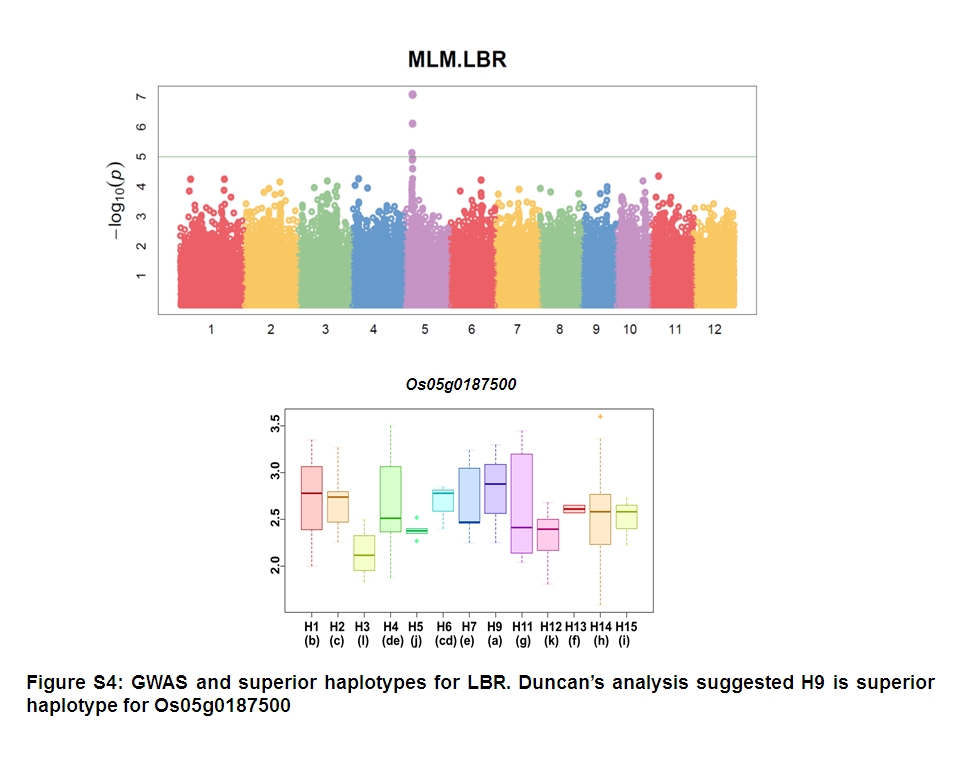


**Figure S4:** GWAS and superior haplotypes for LBR. Duncan’s analysis suggested H9 is superior haplotype for *Os05g0187500*

**
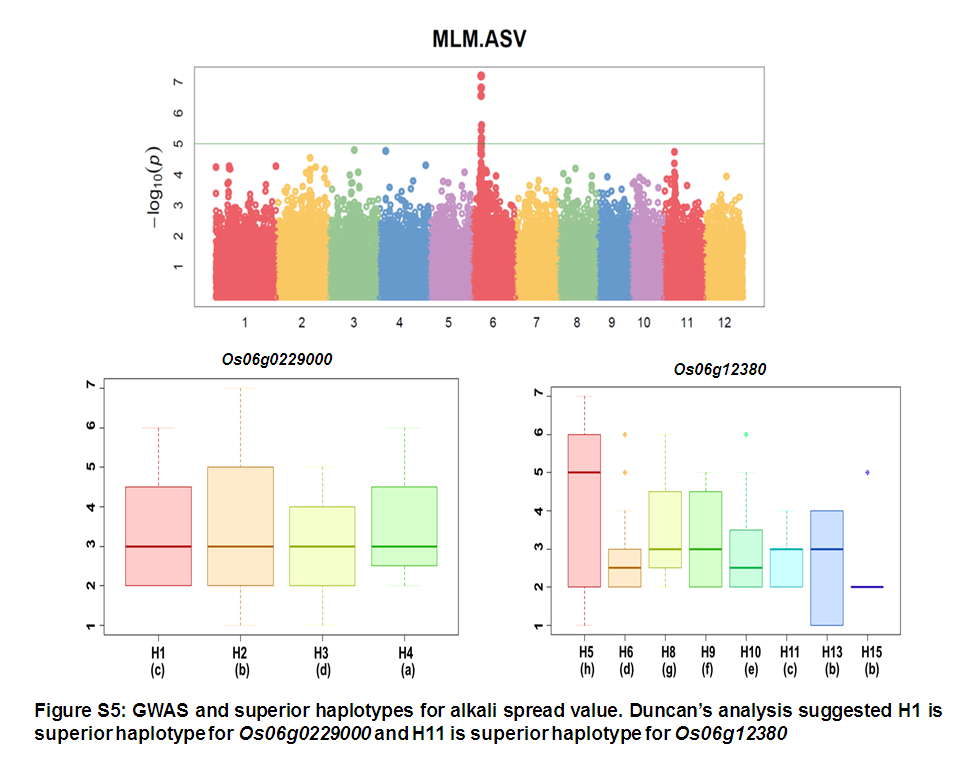
**

**Figure S5:** GWAS and superior haplotypes for alkali spread value. Duncan’s analysis suggested H1 is superior haplotype for *Os06g0229000* and H11 is superior haplotype for *Os06g12380*


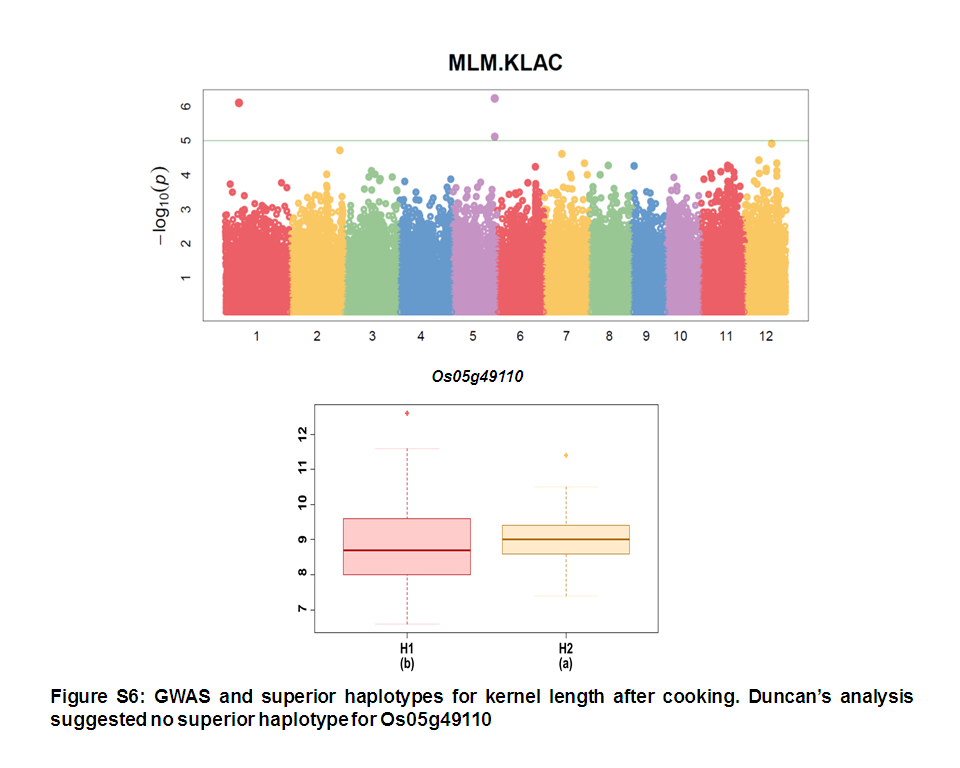


**Figure S6:** GWAS and superior haplotypes for kernel length after cooking. Duncan’s analysis suggested no superior haplotype for *Os05g49110*

***
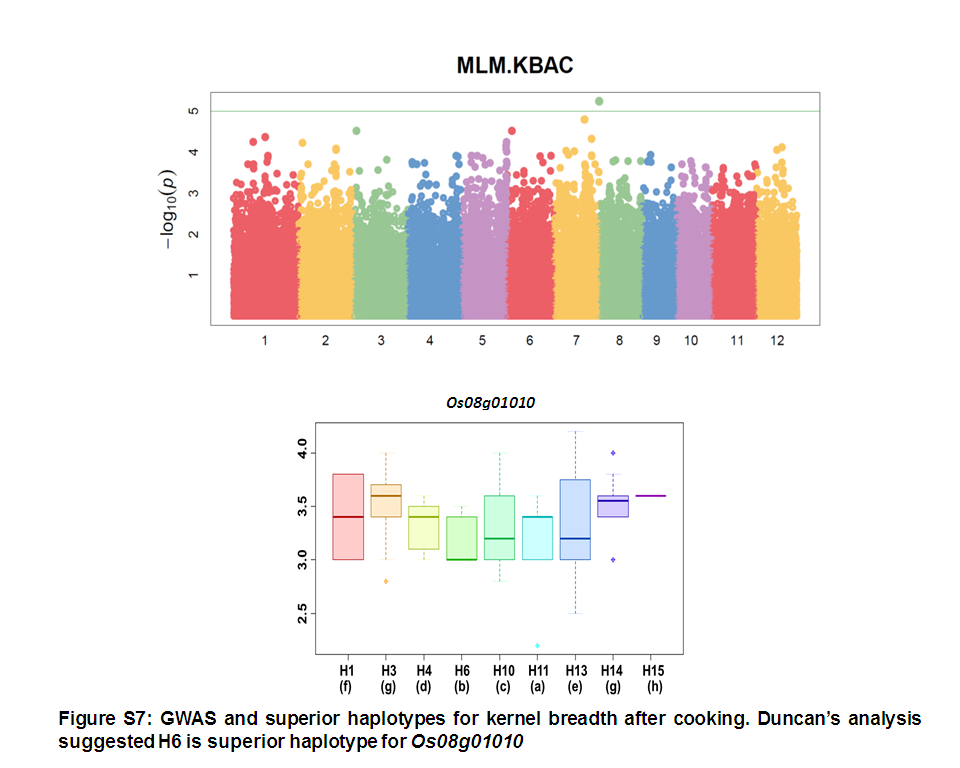
***

**Figure S7:** GWAS and superior haplotypes for kernel breadth after cooking. Duncan’s analysis suggested H6 is superior haplotype for *Os08g01010*

**
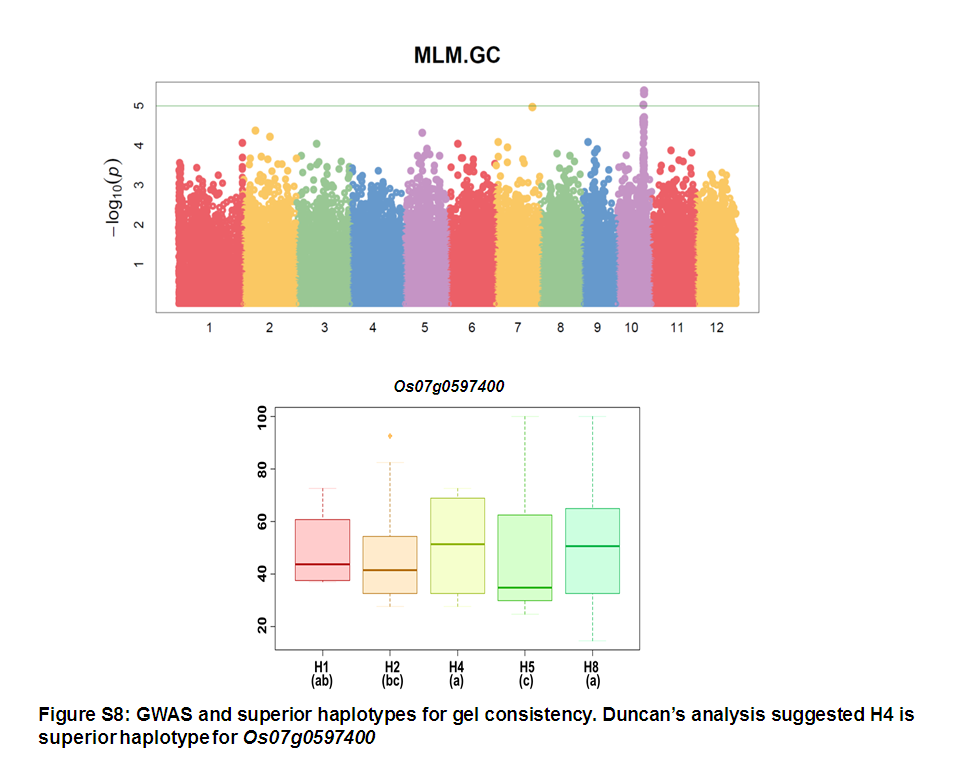
**

**Figure S8:** GWAS and superior haplotypes for gel consistency. Duncan’s analysis suggested H4 is superior haplotype for *Os07g0597400*
